# Impact of Policy Changes Expanding Access to Direct-Acting Antivirals on Hepatitis C Virus–Related Hospitalizations in People With HIV: A Population-Based Study

**DOI:** 10.1093/ofid/ofaf003

**Published:** 2025-01-13

**Authors:** Tony Antoniou, Shaleesa Ledlie, Tianru Wang, Mina Tadrous, Tara Gomes

**Affiliations:** Department of Family and Community Medicine, Unity Health Toronto, Toronto, Ontario, Canada; Li Ka Shing Knowledge Institute, Unity Health Toronto, Toronto, Ontario, Canada; Department of Family and Community Medicine, University of Toronto, Toronto, Canada; ICES, Toronto, Ontario, Canada; Li Ka Shing Knowledge Institute, Unity Health Toronto, Toronto, Ontario, Canada; ICES, Toronto, Ontario, Canada; Leslie Dan Faculty of Pharmacy, University of Toronto, Toronto, Ontario, Canada; ICES, Toronto, Ontario, Canada; ICES, Toronto, Ontario, Canada; Leslie Dan Faculty of Pharmacy, University of Toronto, Toronto, Ontario, Canada; Women's College Research Institute, Toronto, Ontario, Canada; Li Ka Shing Knowledge Institute, Unity Health Toronto, Toronto, Ontario, Canada; ICES, Toronto, Ontario, Canada; Leslie Dan Faculty of Pharmacy, University of Toronto, Toronto, Ontario, Canada; Institute for Health Policy, Management and Evaluation, University of Toronto, Toronto, Ontario, Canada

**Keywords:** direct-acting antivirals, HIV–hepatitis C coinfection, interrupted time series analysis, quasi-experimental methods, hepatitis C virus

## Abstract

**Background:**

The burden of hepatitis C virus (HCV)–related hospitalizations is substantial, particularly among people with HIV and HCV. In Ontario, Canada, use of direct-acting antivirals (DAAs) increased following policies removing fibrosis-stage restrictions and approving of pangenotypic agents in 2017 and 2018, respectively. We examined the impact of expanded DAA access on HCV-related hospitalizations in people with HIV.

**Methods:**

We conducted a population-based study using administrative databases between April 2003 and December 2022. We used segmented negative binomial regression to examine changes in level and trend of quarterly HCV-related hospitalization rates in people with HIV following the policy changes and compared predicted rates in the absence of expanded DAA access with observed rates during this period.

**Results:**

We identified 2943 HCV-related hospitalizations among people with HIV during our study period. Rates of HCV-related hospitalizations were substantially higher among people with HIV than individuals without HIV. In the postintervention period, there was an immediate level increase in the rate of HCV-related hospitalizations (rate ratio, 1.23; 95% CI, 1.18–1.29), followed by a decrease in trend (rate ratio, 0.94 per quarter; 95% CI, .93–.94). We estimated that expanding DAA access was associated with 192 fewer hospitalizations in people with HIV between 2019 and 2022.

**Conclusions:**

Policies expanding DAA access have reduced HCV-related hospitalizations in people with HIV. However, rates were higher relative to those in people without HIV. Further research is needed to identify and address disparities in clinical outcomes among people with HIV and HCV.

## Teaser

In this population-based study, policies that increased access and use of direct-acting antivirals reduced the burden of hepatitis C virus (HCV)–related hospitalizations in people with HIV/HCV. However, rates of these events remained higher relative to people with HCV and no diagnosis of HIV.

Globally, an estimated 2.3 million people with HIV are infected with hepatitis C virus (HCV), among whom the burden of illness is greatest in people who inject drugs and men who have sex with men [1]. Although combination antiretroviral therapy reduces the risk of death and slows HCV-related disease progression in individuals who are coinfected [2, 3], chronic HCV infection remains a leading cause of liver disease and associated mortality in people with HIV/HCV [4, 5].

Fortunately, HCV is now curable with direct-acting antivirals (DAAs), with sustained virologic responses observed in >90% of people with HIV/HCV in clinical trials and observational studies [6–8]. Moreover, the introduction of DAAs has been accompanied by improvements in clinical end points. Specifically, treatment with DAAs was associated with a 3-fold decline in all-cause mortality and a 7-fold decrease in the incidence of liver-related events among participants in the Swiss HIV Cohort Study [9]. Similarly, a significantly reduced risk of death from end-stage liver disease was observed in the Canadian Co-infection Cohort Study between the 2003–2012 and 2013–2017 treatment periods (adjusted hazard ratio, 0.18; 95% CI, .05–.62) [10]. Furthermore, the 10-year risk difference for all-cause mortality was an estimated −3.7% for individuals treated with DAAs in the US Women's Interagency HIV Study and the Multicenter AIDS Cohort Study [11]. Broad and unrestricted access to DAAs also imparts important public health benefits, with declines in the incidence of primary HCV infection and reinfection observed in the International Collaboration on Hepatitis C Elimination in HIV Cohorts consortium [12].

Yet, despite these data, population-based studies examining the impact of DAAs on HCV-related hospitalizations in people with HIV/HCV are lacking [13]. This is important for several reasons. First, a previously published Canadian study found that the burden of hospital admission among people with HIV/HCV is substantial, increasing by 40% per year between 1994 and 2004 [14]. Similar findings have been observed in other studies, with people with HIV/HCV having higher rates of hospitalization and inpatient mortality than those with HIV or HCV alone [15–19]. Second, studies documenting decreased hospitalization rates in people with HCV monoinfection following the advent of DAAs may not be generalizable to the coinfected population because outcomes in these individuals may also be influenced by the independent effects of HIV on liver disease progression [20]. Third, the available evidence associating DAAs with improved outcomes in individuals who are coinfected is derived largely from cohort studies that may not be representative of the entire population of people with HIV/HCV. In contrast, hospitalization records include all persons with disease that is severe enough to warrant hospital admission, including those disengaged from regular care. Finally, in our setting of Ontario, Canada, use of DAAs increased markedly in 2017 and 2018, periods corresponding to the removal of disease-based criteria for DAA treatment and the introduction of drugs with pangenotypic activity, respectively [21]. However, the population-level impact of expanded DAA access on the health system burden of HCV among people with HIV/HCV is unknown. Accordingly, we studied the impact of expanded DAA access on rates of HCV-related hospitalizations in people with HIV in Ontario.

## METHODS

### Setting

We conducted a population-based ecologic time series study examining the impact of DAAs on HCV-related hospitalizations in adults with HIV in Ontario, Canada, between 2003 and 2022. Ontario has the highest number of annual new HIV and HCV cases in Canada and the largest population of Canadians with HIV [22–24]. Our study period encompasses the years when DAAs were first listed on the provincial drug formulary (2015), when disease-based criteria for accessing DAAs were removed (2017), and when pangenotypic drugs were approved (2018) [21].

### Data Sources

We used Ontario's administrative health databases, which were linked by unique encoded identifiers and analyzed at ICES in Toronto, Ontario (https://www.ices.on.ca). ICES is an independent nonprofit research institute whose legal status under section 45 of Ontario's Personal Health Information Protection Act privacy law allows it to collect and analyze health care and demographic data, without consent or review by a research ethics board, for health system evaluation and improvement. We used the Canadian Institute for Health Information's Discharge Abstract Database to identify all HCV-related hospital admissions during our study period. The database contains information regarding all acute hospital admissions in Ontario. We used the Registered Persons Database, the CONTACT database, and the Statistics Canada Postal Code Conversion File to obtain quarterly estimates of the population of Ontario. The Registered Persons Database is a registry of all individuals eligible for publicly funded health insurance, while the CONTACT database contains people’s eligibility status for physician and hospital services in Ontario.

### Study Population

We identified people with HIV using the Ontario HIV Database, an administrative registry derived from a validated case-finding algorithm with a sensitivity of 96.2% (95% CI, 95.2%–97.9%) and a specificity of 99.6% (95% CI, 99.1%–99.8%) for identifying people with HIV in our health databases [25]. We defined individuals with HCV alone as those who had hospitalizations with relevant *ICD-10* diagnostic codes (Supplementary Table 1) but were not included in the Ontario HIV Database.

### Primary Outcome

Our primary outcome was the quarterly rate of HCV-related hospital admissions per 10 000 people with HIV, defined as the number of HCV-related admissions in each quarter divided by the population of Ontario adults with HIV during that period. Specifically, we first defined all admissions with HCV, chronic liver disease, or hepatocellular carcinoma (with or without a recorded liver transplant) as inpatient stays with corresponding *ICD-10* diagnostic codes (Supplementary Table 1). Next, we restricted these records to include only those admissions with an *ICD-10* code of B18.2 and/or B17.1, representing chronic or acute HCV, respectively. This definition has been adapted from past research examining HCV-related hospitalizations in Canada [26, 27]. Finally, we used the Ontario HIV Database to include only those admissions occurring among individuals with a diagnosis of HIV. For those without HIV, we defined the quarterly rate of HCV-related admissions as the number of HCV-related hospitalizations in each quarter among people not diagnosed with HIV, divided by the population of Ontario adults not with HIV during that period. Consequently, for both groups, rates were calculated per the total populations with and without HIV as denominators, reflecting the population-level burden of HCV-related hospitalizations rather than being restricted to persons with confirmed HCV infection.

We compared demographic and clinical characteristics between hospitalized patients with and without diagnosed HIV overall and during the preintervention (2003–2018) and postintervention (2019–2022) periods.

### Statistical Analysis

We used standardized differences to compare demographic and clinical characteristics between hospitalized patients with HIV and those not diagnosed with HIV, with differences >0.1 considered meaningful [28].

In descriptive analyses, we compared mean rates of HCV-related hospitalizations in people with HIV in the pre- (2003–2018) and postintervention (2019–2022) periods. We also derived rate ratios (RRs) comparing HCV-related hospitalizations in people with HIV and those not diagnosed with HIV. For this analysis, we calculated quarterly rates of HCV-related hospitalizations for each group by dividing the number of hospitalizations in a given quarter by the corresponding population, expressed per 10 000 individuals. We then calculated RRs by dividing the hospitalization rate in the HIV/HCV group by the rate in the group not diagnosed with HIV. Exact 95% CIs for the RRs were derived by the Poisson distribution. These calculations were performed for each quarter between 2013 and 2020, including the pre- and postintervention periods. Consequently, the RR reflects the relative burden of HCV-related hospitalizations in the total population of people with HIV as compared with the general population not diagnosed with HIV. To explore and visualize trends in the rate of HCV-related hospitalizations, we applied locally estimated scatterplot smoothing with a smoothing parameter of 0.2. We chose a smoothing parameter of 0.2 to balance the need for retaining short-term fluctuations in the data with highlighting broader trends over time.

In our main analysis, we used negative binomial segmented regression analysis to estimate the level change and the change in trend of HCV-related hospitalizations associated with the availability of DAAs [29, 30]. Although past research has found that DAA use increased markedly following the removal of disease-based restrictions and the availability of pangenotypic drugs, we reasoned that any impact of expanded DAA access on HCV-related hospitalizations would take several months to manifest, given the time needed for patients to access treatment and attain sustained virologic response. We therefore defined the intervention point in all analyses as the first quarter of 2019, approximately 1 year following the approval of pangenotypic drugs. Consequently, our model included a dummy variable denoting the first quarter of 2019, an indicator for time to account for the underlying temporal trend in the data, and an interaction term between time and the dummy variable to estimate the change in HCV-related hospitalization trend following the intervention point. We used the log of the number of people with HIV in each quarter as an offset. Because we detected slight seasonality in our outcome following the decomposition of the time series into seasonal, trend, and irregular components, we added Fourier term coefficients into the model to account for seasonality. We estimated all models using Newey-West standard errors to account for autocorrelation and confirmed the absence of residual autocorrelation through visual inspection of autocorrelation and partial autocorrelation plots (Supplementary Figure 1) [31]. In a sensitivity analysis, we replicated the segmented regression analysis using an intervention date selected with structural break analysis of the time series data set [32].

In a secondary analysis, we determined the expected HCV-related hospitalization rates for the period following the first quarter of 2019 in the absence of policy changes expanding DAA access, using data between 2003 and the fourth quarter of 2018. To do so, we compared the accuracy of several forecast models, including single and double exponential smoothing, Holt-Winters smoothing (additive and multiplicative), and seasonal-trend decomposition with locally estimated scatterplot smoothing (STL) with either exponential smoothing state space or autoregressive integrated moving average (ARIMA) models. We also compared various ARIMA models without STL, selecting the moving average and autoregressive terms using autocorrelation and partial autocorrelation plots, respectively, and selected the best-fitting model using Ljung box tests for white noise and comparisons of the Akaike information criterion [33, 34]. We fit each model over the first 60% of our restricted data set and forecast the models out of sample over the remaining 40% of the observations. We compared the accuracy of forecasts from each model with the actual values using the mean absolute percentage error. We used the most accurate model to generate in-sample forecasts for the period following the first quarter of 2019. We determined the relative percentage changes between the observed and predicted HCV-related hospitalization rates and estimated associated 95% CIs using the Poisson distribution. All analyses were completed with SAS version 9.4 (SAS Institute), Stata version 18.0 (StataCorp LLC), and R Studio.

## RESULTS

During our 20-year study period, we identified 1288 individuals with HIV and 24 832 not diagnosed with HIV with at least 1 HCV-related hospitalization (Table 1). When compared with those without HIV, those with HIV were more likely to be male (75.3% vs 63.3%; standardized difference, 0.26) and have a mental health diagnosis in the year preceding hospitalization (23.5% vs 17.0%; standardized difference, 0.16). The proportion hospitalized with hepatocellular carcinoma (10.2% vs 3.3%; standardized difference, 0.28) and fibrosis or cirrhosis (28.4% vs 14.4%; standardized difference, 0.35) was higher among those not diagnosed with HIV. Overall, the characteristics of hospitalized patients with HIV and those without HIV did not change appreciably between the pre- and postintervention periods.

**Table 1.** Demographic and Clinical Characteristics of People With and Without HIV and an HCV-Related Hospitalization, 2003–2022

	Preintervention (2003–2018)					Postintervention (2019–2022)					Overall (2003–2022)				
	With HIV		Without HIV			With HIV		Without HIV			With HIV		Without HIV		
Characteristic	No.	%	No.	%	Standardized Difference	No.	%	No.	%	Standardized Difference	No.	%	No.	%	Standardized Difference
**Demographics**															
No. of individuals	1083		21 113			351		5109			1288		24 832		
Age, y, mean (SD)	46.6 (10.1)		54.4 (14.0)		0.64	49.8 (12.0)		53.6 (15.0)		0.28	47.5 (10.6)		54.4 (14.2)		0.55
Sex															
Women	261	24.1	7785	36.9	0.28	103	29.3	1903	37.3	0.17	318	24.7	9120	36.7	0.26
Men	822	75.9	13 328	63.1	0.28	248	70.7	3206	62.8	0.17	970	75.3	15 712	63.3	0.26
Neighborhood income quintile															
1	482	44.5	7989	37.8	0.14	171	48.7	2254	44.1	0.09	583	45.3	9626	38.8	0.13
2	227	21.0	4627	21.9	0.02	71	20.2	1145	22.4	0.05	277	21.5	5447	21.9	0.01
3	159	14.7	3466	16.4	0.05	48	13.7	724	14.2	0.01	186	14.4	3982	16.0	0.04
4	94	8.7	2688	12.7	0.13	17	4.8	510	10.0	0.20	97	7.5	3077	12.4	0.16
5	94	8.7	2112	10.0	0.05	31	8.8	382	7.5	0.05	113	8.8	2394	9.6	0.03
Missing	27	2.5	231	1.1	0.11	13	3.7	94	1.8	0.11	32	2.5	306	1.2	0.09
Location of residence															
Urban	1001	92.4	19 149	90.7	0.06	325	92.6	4505	88.2	0.15	1192	92.6	22 360	90.1	0.09
Rural	62	5.7	1844	8.7	0.12	13	3.7	514	10.1	0.25	71	5.5	2276	9.2	0.14
Missing	20	1.9	120	0.57	0.12	13	3.7	90	1.8	0.12	25	1.9	196	0.79	0.10
**Liver diagnosis at time of hospitalization**															
Malignant neoplasm of liver and intrahepatic bile duct	32	3.0	2194	10.4	0.30	11	3.1	417	8.2	0.22	42	3.3	2535	10.2	0.28
Chronic viral hepatitis	1074	99.2	20 852	98.8	0.04	349	99.4	5007	98.0	0.13	1278	99.2	24 488	98.6	0.06
Fibrosis and cirrhosis of liver	162	15.0	6207	29.4	0.35	37	10.5	1165	22.8	0.33	186	14.4	7051	28.4	0.35
Other disease of liver	105	9.7	3711	17.6	0.23	16	4.6	667	13.1	0.30	114	8.9	4233	17.1	0.25
**Mental health diagnosis identified in year prior to admission**															
Any mental health and additional diagnosis	255	23.6	3368	16.0	0.19	102	29.1	1204	23.6	0.13	303	23.5	4228	17.0	0.16
Anxiety disorder	36	3.3	547	2.6	0.04	≤10	≤3.0	88	1.7	0.02	38	3.0	596	2.4	0.03
Deliberate self-harm	50	4.6	819	3.9	0.04	17	4.8	252	4.9	0.00	54	4.2	993	4.0	0.01
Mood disorder	49	4.5	617	2.9	0.08	≤5	≤2.0	109	2.1	…	43	3.3	667	2.7	0.04
Schizophrenia	35	3.2	331	1.6	0.11	≤10	≤3.0	134	2.6	0.00	42	3.3	430	1.7	0.1
Substance use disorder	172	15.9	2301	10.9	0.15	84	23.9	971	19.0	0.12	214	16.6	3008	12.1	0.13
Trauma/stressor-related disorders	17	1.6	121	0.57	0.10	13	3.7	159	3.1	0.03	20	1.6	253	1.0	0.05
Other mental health diagnoses^a^	22	2.0	211	1.0	0.08	≤5	≤2.0	87	1.7	…	22	1.7	271	1.1	0.05

Some cells are suppressed because of their small sample size (n ≤ 5), which cannot be reported per privacy regulations.

Abbreviation: HCV, hepatitis C virus.

^a^Including personality disorders, obsessive-compulsive disorder, and related disorders.

**Figure 1.**
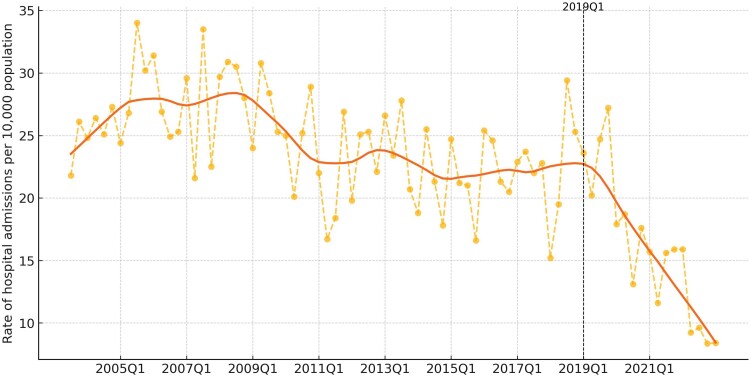
Hepatitis C virus–related hospitalizations among people with HIV, 2003 to 2022.

We identified 2943 HCV-related hospitalizations among people with HIV during our study period, with rates declining following the intervention period (Figure 1). The mean rates of HCV-related hospitalizations in the periods preceding (2003–2018) and following (2019–2022) the intervention point were 24.6 (SD, 4.1) and 15.6 (SD, 5.5) per 10 000 individuals with HIV, respectively (*P* < .001). The RR for the postintervention period as compared with the preintervention period was 0.64 (95% CI, .58–.70). Rates of HCV-related hospitalizations were higher among people with HIV than those not diagnosed with HIV at all periods, including the postintervention period (Supplementary Figure 2). Specifically, HCV-related hospitalization RRs for people with HIV relative to those without HIV ranged from 39.0 (95% CI, 23.8–60.6) to 68.7 (95% CI, 51.6–89.9) during the postintervention period (Supplementary Table 2).

In our main analysis, there was an increase in the rate of HCV-related hospitalizations immediately following the postintervention period (RR, 1.23; 95% CI, 1.18–1.29). However, HCV-related hospitalizations declined steadily after this initial increase, with a relative decrease in trend of 0.94 per quarter (95% CI, .93–.94). In our sensitivity analysis, a structural break in the time series was identified during the first quarter of 2018. Using this date rather than the first quarter of 2019 as the intervention point resulted in a similar change in the quarterly HCV-related hospitalization trend (RR, 0.95; 95% CI, .94–.95).

**Figure 2.**
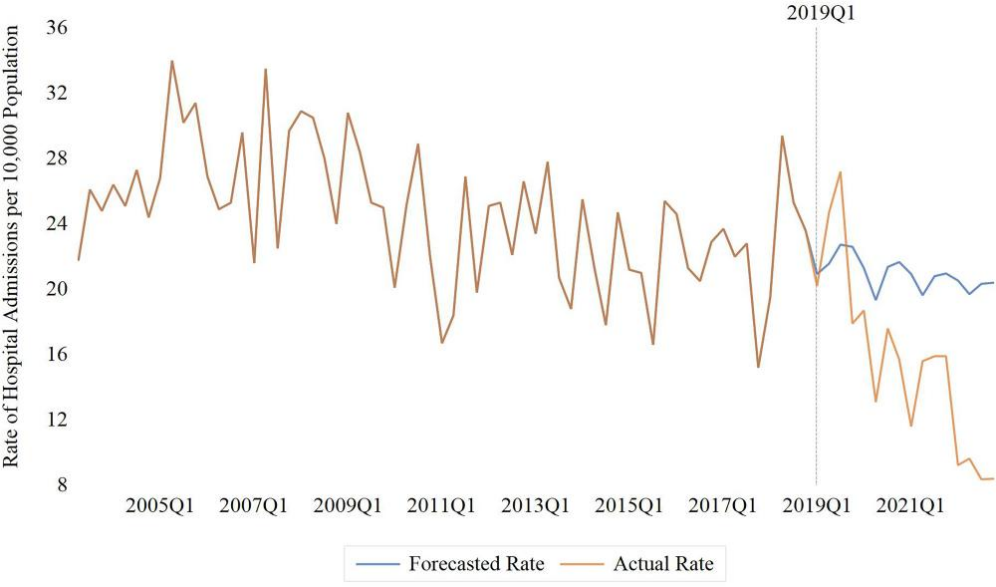
Observed vs forecasted rates of hepatitis C virus–related hospitalizations among people with HIV 1 year following expanded direct-acting antiviral access. Dotted line represents intervention point (i.e. first quarter of 2019).

In our secondary analysis, a seasonal ARIMA (3,1,0)(0,0,2,4) model had the lowest mean absolute percentage error of all models tested and was used to generate forecasts for the postintervention period (Supplementary Figure 3 for autocorrelation and partial autocorrelation residual plots). Overall, the observed mean rate of HCV-related hospitalizations between 2019 and 2022 was 25.8% lower than predicted (15.6 vs 20.9 hospitalizations per 10 000; RR, 0.74; 95% CI, .66–.83), corresponding to 192 fewer admissions for HCV-related liver disease during this period. Hospitalization rates were consistently below forecasted rates at all points including and following the fourth quarter of 2019, with the most pronounced differences observed in 2022 (Supplementary Table 3, Figure 2).

## DISCUSSION

In our population-based study, we observed an initial increase in HCV-related hospitalizations among people with HIV following policy changes expanding DAA access, followed by a steady decline in these events in the ensuing quarters. Moreover, hospitalization rates were lower than those forecasted in the absence of these policy changes. However, rates of HCV-related hospitalizations were substantially higher among people with HIV as compared with individuals without HIV throughout the entire study period.

Our study builds on past research demonstrating that policy changes intended to increase DAA access facilitated treatment uptake among Ontario residents with HCV. Specifically, a population-based study of people with publicly funded drug coverage in Ontario found that DAA use increased following policy changes in 2017 and 2018 that removed disease-based criteria for accessing treatment and added pangenotypic agents to the provincial drug formulary, with the greatest peak in use occurring in early 2017 [21]. Similarly, HCV treatment rates increased 1.8-fold following the 2017 removal of fibrosis-stage restrictions among participants in the Canadian Co-infection Cohort Study, which includes Ontario residents [35]. The impact was more pronounced among people who inject drugs, with a 3.8-fold increase in treatment rates following the removal of fibrosis-stage restrictions [35]. Our findings provide evidence for the health-system and population-level impacts of these policy changes among people with HIV/HCV, with a reduced trend in HCV-related hospitalizations observed after allowing at least a 1-year lag for the measures to take effect. However, a sensitivity analysis indicated that the decline in HCV-related hospitalizations occurred earlier than our prespecified change point, beginning approximately 1 year following the 2017 removal of disease-based criteria for funding. This finding is consistent with results of earlier research demonstrating large increases in DAA treatment uptake following this period [21, 35] and may reflect a “warehousing effect” whereby clinicians waited until treatment access was expanded before treating patients who regularly engage in care and were perceived as most likely to adhere to treatment [35, 36].

This phenomenon may explain in part the initial step increase in HCV-related hospitalizations observed following the intervention period. Specifically, this finding may reflect a backlog of patients with advanced HCV-related disease and higher risk of hospitalization who had been warehoused until DAAs became more widely accessible. Further research is required to account for this finding.

Our work contributes to the emerging body of literature demonstrating the impact of DAAs on hospitalizations in people with HCV. Specifically, a retrospective study of 378 people with HCV and cirrhosis found that DAA treatment was associated with a 64.3% reduction in liver-related hospitalizations [37]. In a study of 1073 people with HCV, those responding to DAA treatment had reduced rates of hospitalization for decompensated cirrhosis (hazard ratio, 0.14; 95% CI, .05–.39) and hepatocellular carcinoma (hazard ratio, 0.17; 95% CI, .04–.79) [38]. Furthermore, a Canadian study of hospitalization records between 2004 and 2016 revealed that HCV-related hospitalizations declined following an increase in use of DAAs in 2015, with the most pronounced effects observed among patients born prior to 1960 [39]. A subsequent study with follow-up extended to 2020 reported similar results, with decreases in HCV-related hospitalizations occurring in 2016 and 2018 [27]. Our findings build on this work by providing the first empirical evidence indicating that expanded access to DAAs reduced HCV-related hospitalizations in people with HIV/HCV, a population that prior research has identified as being at higher risk for HCV-related hospitalization than people with HCV alone [15–19]. Further research is needed to determine if these results are sustained, particularly as rates of reinfection increase [40].

Our findings have implications for the health of people with HIV and HCV. Most notably, we observed substantially higher rates of HCV-related hospitalization relative to individuals not diagnosed with HIV throughout the entire study period, highlighting an ongoing vulnerability of people with HIV/HCV to severe liver disease. This may reflect incomplete resolution of inflammatory and profibrogenic stimuli in persons with HIV/HCV treated with DAAs relative to those with HCV alone, even after attaining a sustained virologic response [41]. In addition, HIV itself may cause or potentiate fibrosis through several profibrotic pathways independent of coinfection with hepatitis viruses [20, 41]. It is therefore possible that people with HIV/HCV require ongoing assessment of fibrosis progression and surveillance for hepatocellular carcinoma after successful treatment with DAAs [41]. Our findings may also reflect disparities in treatment access, with past research showing longer median times between a first clinic evaluation for HCV and DAA initiation in people with HIV/HCV relative to people with HCV alone (ie, 298 vs 161 days) [42]. In addition, DAA access among people with HIV/HCV is not equitable. Specifically, a study examining treatment uptake following the removal of fibrosis restrictions found that persons of Indigenous ethnicity, people who inject drugs, individuals experiencing homelessness, and those disengaged from care were more likely to remain HCV RNA positive and therefore still require HCV treatment [35]. Research is needed to develop community-informed models of care that remove structural and social barriers to accessing DAA treatment among people with HIV/HCV.

Our study has some limitations. First, our administrative health databases lack important clinical and laboratory data, including HIV and HCV exposure categories, HCV genotype, and extent of fibrosis. We were therefore unable to assess trends among specific subgroups of people with HIV/HCV. We also did not have access to HIV and HCV laboratory testing. However, we used a validated administrative registry with high sensitivity and specificity for identifying people with HIV. Similarly, we did not have access to accurate estimates of DAA use, precluding an assessment of the role of treatment disparities in our findings. Future research is needed to identify specific subgroups of people with HIV/HCV who have not accessed treatment and the reasons for treatment inequity. Second, we were unable to assess trends in hepatocellular carcinoma because of the small number of these events. Third, our findings are based on a population with publicly funded access to physician services and hospital care. It is possible that our findings may not be generalizable to other contexts. Fourth, our definition of HCV-related hospitalizations has not been validated such that the sensitivity and specificity of this algorithm for capturing such admissions are unknown. Yet, we used a definition consistent with prior national and provincial studies, which allows for comparison with past research. Because the definition required that all records include a diagnosis code of acute or chronic HCV infection, it is possible that we are underestimating the number of HCV-related hospitalizations, as these diagnostic codes may not have been used for all such admissions. Conversely, it is possible that some admissions were misclassified as HCV related when HCV was not the primary cause of hospitalization. Still, this is unlikely, as our definition restricted the outcome to admissions for liver disease co-occurring with diagnoses of acute or chronic hepatitis C. Finally, our follow-up was limited to the first 4 years following the policy changes facilitating DAA access. It is unknown if the impact of these changes would be sustained with longer follow-up, particularly as the incidence of reinfection increases [40].

In summary, our study suggests that policies intended to improve DAA access and the associated increase in DAA use have reduced HCV hospitalization rates among people with HIV. However, rates were markedly higher relative to those observed among individuals not diagnosed with HIV, reflecting the continued burden of severe liver disease in people with HIV and HCV. Further research is needed to identify and address disparities in DAA treatment access and clinical outcomes among people with HIV and HCV.

## Supplementary Material

ofaf003_Supplementary_Data

## Supplementary Data

Supplementary materials are available at *Open Forum Infectious Diseases* online. Consisting of data provided by the authors to benefit the reader, the posted materials are not copyedited and are the sole responsibility of the authors, so questions or comments should be addressed to the corresponding author.

## Notes

***Acknowledgments*.** Parts of this material are based on data and information compiled and provided by the Ontario Ministry of Health, Ontario Health, and the Canadian Institute for Health Information. This document used data adapted from the Statistics Canada Postal Code^OM^ Conversion File, which is based on data licensed from Canada Post Corporation, and/or data adapted from the Ontario Ministry of Health Postal Code Conversion File, which contains data copied under license from Canada Post Corporation and Statistics Canada. T. G. holds a Canada Research Chair in Drug Policy Research and Evaluation.

***Author contributions*.** T. A., T. W., S. L., M. T., and T. G. conceptualized and designed the study and were involved in the interpretation of the data. T. A. conducted the analyses and drafted the initial manuscript. All authors critically reviewed the manuscript for intellectual content and revised the manuscript. All authors approved the final manuscript as submitted and agree to be accountable for all aspects of the work in ensuring that questions related to the accuracy or integrity of any part of the work are appropriately investigated and resolved.

***Data availability*.** The data set from this study is held securely in coded form at ICES. While legal data-sharing agreements between ICES and data providers (eg, health care organizations and government) prohibit ICES from making the data set publicly available, access may be granted to those who meet prespecified criteria for confidential access, available at www.ices.on.ca/DAS.

***Disclaimer.*** The analyses, conclusions, opinions, and statements expressed herein are solely those of the authors and do not reflect those of the funding or data sources; no endorsement is intended or should be inferred.

***Financial support.*** This work was supported by ICES, which is funded by an annual grant from the Ontario Ministry of Health and the Ministry of Long-term Care. This study was funded by the Ministry of Health (grant 0691).
