# Supplementary material for: Impact of Policy Changes Expanding Access to Direct-Acting Antivirals on Hepatitis C Virus–Related Hospitalizations in People With HIV: A Population-Based Study
Source: Open Forum Infect Dis. 2025 Jan 13;12(1):ofaf003. doi: 10.1093/ofid/ofaf003 (PMC11773189; doi:10.1093/ofid/ofaf003)

**Supplemental Table 1: International Classification of Diseases, 10th revision (ICD-10) Diagnostic Codes for Main Outcome**

| **ICD-10 Code** | **ICD-10 Code Description** |
| --- | --- |
| B18 | Chronic viral hepatitis |
| B18.2 | Chronic viral hepatitis C |
| B17.1 | Acute hepatitis C |
| B17.9 | Acute viral hepatitis, unspecified |
| B19 | Unspecified viral hepatitis |
| C22 | Malignant neoplasm of liver and intrahepatic bile duct |
| I85 | Esophageal varices |
| I86.4 | Gastric varices |
| I98.2 | Oesophageal varices without bleeding in diseases classified elsewhere |
| I98.3 | Oesophageal varices with bleeding in diseases classified elsewhere |
| K70 | Alcoholic liver disease |
| K71 | Toxic liver disease |
| K72 | Hepatic failure, not elsewhere classified |
| K73 | Chronic hepatitis, not elsewhere classified |
| K74 | Fibrosis and cirrhosis of liver |
| K75 | Other inflammatory liver disease |
| K76 | Other disease of liver |
| K77 | Liver disorders in diseases classified elsewhere |
| K91.83 | Postprocedural hepatorenal syndrome |
| R18 | Ascites |

**Supplemental Table 2: HCV-related hospitalization rates (per 10,000 individuals) for people with HIV and individuals not diagnosed with HIV, and rate ratios comparing the two groups**

| **Year/quarter** | **HCV-Related Hospitalizations per 10,000 People with HIV (95% CI)** | **HCV-Related Hospitalizations per 10,000 Individuals Not Diagnosed with HIV (95% CI)** | **Rate Ratio of HCV-Related Hospitalizations: People with HIV vs. Individuals Not Diagnosed with HIV (95% CI)** |
| --- | --- | --- | --- |
| 2003q2 | 21.8 (13.8-32.7) | 0.35 (0.32-0.38) | 62.3 (39.1-94.8) |
| 2003q3 | 26.1 (17.4-37.8) | 0.39 (0.35-0.42) | 67.7 (44.5-99.2) |
| 2003q4 | 24.8 (16.4-36.1) | 0.43 (0.39-0.47) | 58.0 (37.9-85.4) |
| 2004q1 | 26.4 (17.7-37.9) | 0.43 (0.39-0.46) | 61.9 (41.1-90.0) |
| 2004q2 | 25.1 (16.7-36.3) | 0.36 (0.33-0.40) | 69.0 (45.3-101.1) |
| 2004q3 | 27.3 (18.6-38.8) | 0.37 (0.34-0.40) | 74.3 (49.9-107.0) |
| 2004q4 | 24.4 (16.2-35.2) | 0.39 (0.35-0.42) | 62.8 (41.3-91.9) |
| 2005q1 | 26.8 (18.2-38.0) | 0.43 (0.39-0.46) | 62.9 (42.3-90.4) |
| 2005q2 | 34.0 (24.3-46.3) | 0.42 (0.38-0.45) | 81.6 (57.6-112.6) |
| 2005q3 | 30.2 (21.1-41.7) | 0.46 (0.42-0.50) | 65.8 (45.7-92.2) |
| 2005q4 | 31.4 (22.3-43.1) | 0.41 (0.38-0.45) | 76.2 (53.3-106.0) |
| 2006q1 | 26.9 (18.6-37.8) | 0.44 (0.40-0.48) | 61.5 (41.9-87.3) |
| 2006q2 | 24.9 (16.9-35.3) | 0.47 (0.43-0.51) | 53.1 (35.8-76.2) |
| 2006q3 | 25.3 (17.3-35.7) | 0.47 (0.44-0.51) | 53.4 (36.2-76.2) |
| 2006q4 | 29.6 (21.0-40.6) | 0.52 (0.48-0.56) | 57.0 (40.0-79.1) |
| 2007q1 | 21.6 (14.3-31.2) | 0.49 (0.46-0.53) | 43.7 (28.8-63.7) |
| 2007q2 | 33.5 (24.4-45.0) | 0.45 (0.41-0.49) | 74.5 (53.5-101.3) |
| 2007q3 | 22.5 (15.2-32.1) | 0.51 (0.47-0.55) | 44.6 (29.8-64.3) |
| 2007q4 | 29.7 (21.2-40.4) | 0.49 (0.46-0.53) | 60.2 (42.6-83.0) |
| 2008q1 | 30.9 (22.3-41.8) | 0.50 (0.46-0.54) | 61.9 (44.1-84.6) |
| 2008q2 | 30.5 (22.0-41.2) | 0.49 (0.45-0.53) | 62.4 (44.5-85.3) |
| 2008q3 | 28.0 (19.9-38.2) | 0.50 (0.46-0.54) | 55.8 (39.3-77.2) |
| 2008q4 | 24.0 (16.6-33.5) | 0.47 (0.44-0.51) | 50.8 (34.8-71.7) |
| 2009q1 | 30.8 (22.4-41.3) | 0.46 (0.42-0.50) | 67.0 (48.2-91.1) |
| 2009q2 | 28.4 (20.4-38.5) | 0.46 (0.42-0.49) | 62.2 (44.2-85.4) |
| 2009q3 | 25.3 (17.8-34.8) | 0.50 (0.47-0.54) | 50.3 (35.1-70.1) |
| 2009q4 | 25.0 (17.6-34.4) | 0.45 (0.41-0.48) | 56.0 (39.0-78.0) |
| 2010q1 | 20.1 (13.6-28.7) | 0.48 (0.45-0.52) | 41.6 (27.9-60.0) |
| 2010q2 | 25.2 (17.8-34.6) | 0.51 (0.47-0.55) | 49.4 (34.7-68.6) |
| 2010q3 | 28.9 (21.0-38.8) | 0.54 (0.50-0.58) | 53.9 (38.8-73.1) |
| 2010q4 | 22.0 (15.3-30.8) | 0.54 (0.50-0.58) | 41.0 (28.1-57.8) |
| 2011q1 | 16.7 (10.9-24.4) | 0.51 (0.48-0.55) | 32.5 (21.1-48.1) |
| 2011q2 | 18.4 (12.3-26.4) | 0.51 (0.48-0.55) | 35.7 (23.8-51.8) |
| 2011q3 | 26.9 (19.5-36.3) | 0.56 (0.52-0.60) | 48.2 (34.6-65.6) |
| 2011q4 | 19.8 (13.5-27.9) | 0.55 (0.51-0.59) | 36.1 (24.5-51.4) |
| 2012q1 | 25.1 (18.0-34.0) | 0.50 (0.46-0.54) | 50.0 (35.5-68.6) |
| 2012q2 | 25.3 (18.3-34.2) | 0.56 (0.52-0.60) | 45.0 (32.2-61.5) |
| 2012q3 | 22.1 (15.5-30.4) | 0.54 (0.50-0.58) | 41.1 (28.7-57.1) |
| 2012q4 | 26.6 (19.4-35.5) | 0.56 (0.52-0.60) | 47.6 (34.4-64.3) |
| 2013q1 | 23.4 (16.7-31.9) | 0.56 (0.52-0.60) | 42.0 (29.8-57.8) |
| 2013q2 | 27.8 (20.5-36.9) | 0.59 (0.55-0.63) | 47.4 (34.7-63.5) |
| 2013q3 | 20.7 (14.5-28.6) | 0.61 (0.57-0.66) | 33.8 (23.5-47.2) |
| 2013q4 | 18.8 (12.9-26.4) | 0.62 (0.58-0.66) | 30.4 (20.8-43.0) |
| 2014q2 | 21.3 (15.1-29.2) | 0.57 (0.53-0.61) | 37.5 (26.3-51.9) |
| 2014q3 | 17.8 (12.2-25.1) | 0.54 (0.50-0.58) | 33.0 (22.4-47.0) |
| 2014q4 | 24.7 (18.0-33.0) | 0.53 (0.49-0.57) | 46.6 (33.7-63.0) |
| 2015q1 | 21.2 (15.1-29.0) | 0.54 (0.51-0.59) | 39.0 (27.5-53.9) |
| 2015q2 | 21.0 (15.0-28.7) | 0.53 (0.49-0.57) | 40.0 (28.2-55.3) |
| 2015q3 | 16.6 (11.3-23.5) | 0.52 (0.48-0.56) | 32.1 (21.7-46.0) |
| 2015q4 | 25.4 (18.7-33.7) | 0.51 (0.47-0.55) | 50.0 (36.5-67.1) |
| 2016q1 | 24.6 (18.1-32.7) | 0.48 (0.45-0.52) | 51.0 (37.1-68.5) |
| 2016q2 | 21.3 (15.3-28.8) | 0.45 (0.41-0.48) | 47.7 (33.9-65.5) |
| 2016q3 | 20.5 (14.7-27.9) | 0.46 (0.43-0.50) | 44.7 (31.6-61.5) |
| 2016q4 | 22.9 (16.7-30.6) | 0.47 (0.43-0.50) | 49.1 (35.5-66.5) |
| 2017q1 | 23.7 (17.4-31.5) | 0.43 (0.40-0.47) | 55.3 (40.2-74.4) |
| 2017q2 | 22.0 (16.0-29.5) | 0.44 (0.40-0.47) | 50.3 (36.2-68.4) |
| 2017q3 | 22.8 (16.7-30.4) | 0.44 (0.40-0.47) | 52.4 (37.9-70.7) |
| 2017q4 | 15.2 (10.3-21.6) | 0.38 (0.35-0.42) | 39.7 (26.7-57.0) |
| 2018q1 | 19.5 (13.9-26.5) | 0.40 (0.36-0.43) | 49.3 (34.8-68.0) |
| 2018q2 | 29.4 (22.5-37.7) | 0.47 (0.44-0.51) | 62.3 (47.1-81.1) |
| 2018q3 | 25.3 (18.9-33.0) | 0.49 (0.45-0.53) | 51.9 (38.5-68.6) |
| 2018q4 | 23.6 (17.6-31.2) | 0.49 (0.45-0.53) | 48.3 (35.5-64.4) |
| 2019q1 | 20.2 (14.6-27.2) | 0.46 (0.42-0.49) | 44.1 (31.6-60.1) |
| 2019q2 | 24.7 (18.5-32.3) | 0.41 (0.38-0.45) | 59.9 (44.3-79.4) |
| 2019q3 | 27.2 (20.7-35.1) | 0.40 (0.37-0.43) | 68.7 (51.6-89.9) |
| 2019q4 | 17.9 (12.7-24.4) | 0.41 (0.38-0.44) | 43.8 (30.8-60.6) |
| 2020q1 | 18.7 (13.4-25.4) | 0.33 (0.31-0.37) | 56.1 (39.7-77.2) |
| 2020q2 | 13.1 (8.8-18.8) | 0.31 (0.28-0.34) | 42.3 (28.0-61.5) |
| 2020q3 | 17.6 (12.5-24.0) | 0.33 (0.30-0.36) | 53.9 (37.9-74.8) |
| 2020q4 | 15.7 (10.9-21.8) | 0.33 (0.30-0.36) | 47.8 (32.9-67.4) |
| 2021q1 | 11.6 (7.6-17.0) | 0.30 (0.27-0.33) | 39.2 (25.3-58.2) |
| 2021q2 | 15.6 (10.8-21.6) | 0.32 (0.29-0.35) | 49.3 (33.9-69.5) |
| 2021q3 | 15.9 (11.2-22.1) | 0.28 (0.25-0.31) | 56.8 (39.3-80.0) |
| 2021q4 | 15.9 (11.1-21.9) | 0.27 (0.24-0.30) | 59.0 (40.7-83.0) |
| 2022q1 | 9.2 (5.7-14.1) | 0.24 (0.21-0.26) | 39.0 (23.8-60.6) |
| 2022q2 | 9.6 (6.0-14.6) | 0.21 (0.19-0.23) | 46.4 (28.6-71.5) |
| 2022q3 | 8.3 (5.0-13.0) | 0.22 (0.20-0.24) | 38.3 (22.8-60.8) |
| 2022q4 | 8.4 (5.0-13.1) | 0.21 (0.18-0.23) | 40.7 (24.2-64.7) |

**Supplemental Table 3: Forecasted and Actual Rates of HCV-Related Hospitalizations Among People with HIV per 10,000 population with HIV**

| **Year/Quarter** | **Projected Rate of Hospitalization (per 10000 population)** | **Actual Rate of Hospitalization (per 10000 population)** | **Relative Percent Change: Actual vs. Projected Hospitalization Rate (95% Confidence Interval)** |
| --- | --- | --- | --- |
| 2019q1 | 20.9 | 20.20 | -2.3% (-43.3% to 38.8%) |
| 2019q2 | 21.6 | 24.70 | 15.2% (-30.3% to 60.7%) |
| 2019q3 | 22.7 | 27.20 | 20.4% (-25.2% to 66.0%) |
| 2019q4 | 22.6 | 17.90 | -20.4% (-53.9% to 13.1%) |
| 2020q1 | 21.3 | 18.70 | -10.9% (-48.4% to 26.7%) |
| 2020q2 | 19.3 | 13.10 | -31.0% (-63.6% to 1.7%) |
| 2020q3 | 21.3 | 17.60 | -17.0% (-52.2% to 18.2%) |
| 2020q4 | 21.6 | 15.70 | -27.1% (-58.8% to 4.7%) |
| 2021q1 | 20.9 | 11.60 | -43.5% (-70.7% to -16.3%) |
| 2021q2 | 19.6 | 15.60 | -20.5% (-55.8% to 14.9%) |
| 2021q3 | 20.8 | 15.90 | -21.7% (-55.9% to 12.4%) |
| 2021q4 | 20.9 | 15.90 | -23.4% (-56.7% to 9.8%) |
| 2022q1 | 20.5 | 9.23 | -54.3% (-77.9% to -30.8%) |
| 2022q2 | 19.7 | 9.63 | -50.0% (-75.6% to -24.4%) |
| 2022q3 | 20.3 | 8.35 | -58.7% (-80.8% to -36.6%) |
| 2022q4 | 20.4 | 8.39 | -58.7% (-80.8% to -36.6%) |

**Supplementary Figure 1: Autocorrelation and partial autocorrelation plots for interrupted time series analysis of HCV-related hospitalizations**

**Supplemental Figure 2: Hepatitis C Virus (HCV)-Related Hospitalization Rate Ratios: Rates per 10,000 People with HIV Compared to Rates per 10,000 People Without HIV**

**
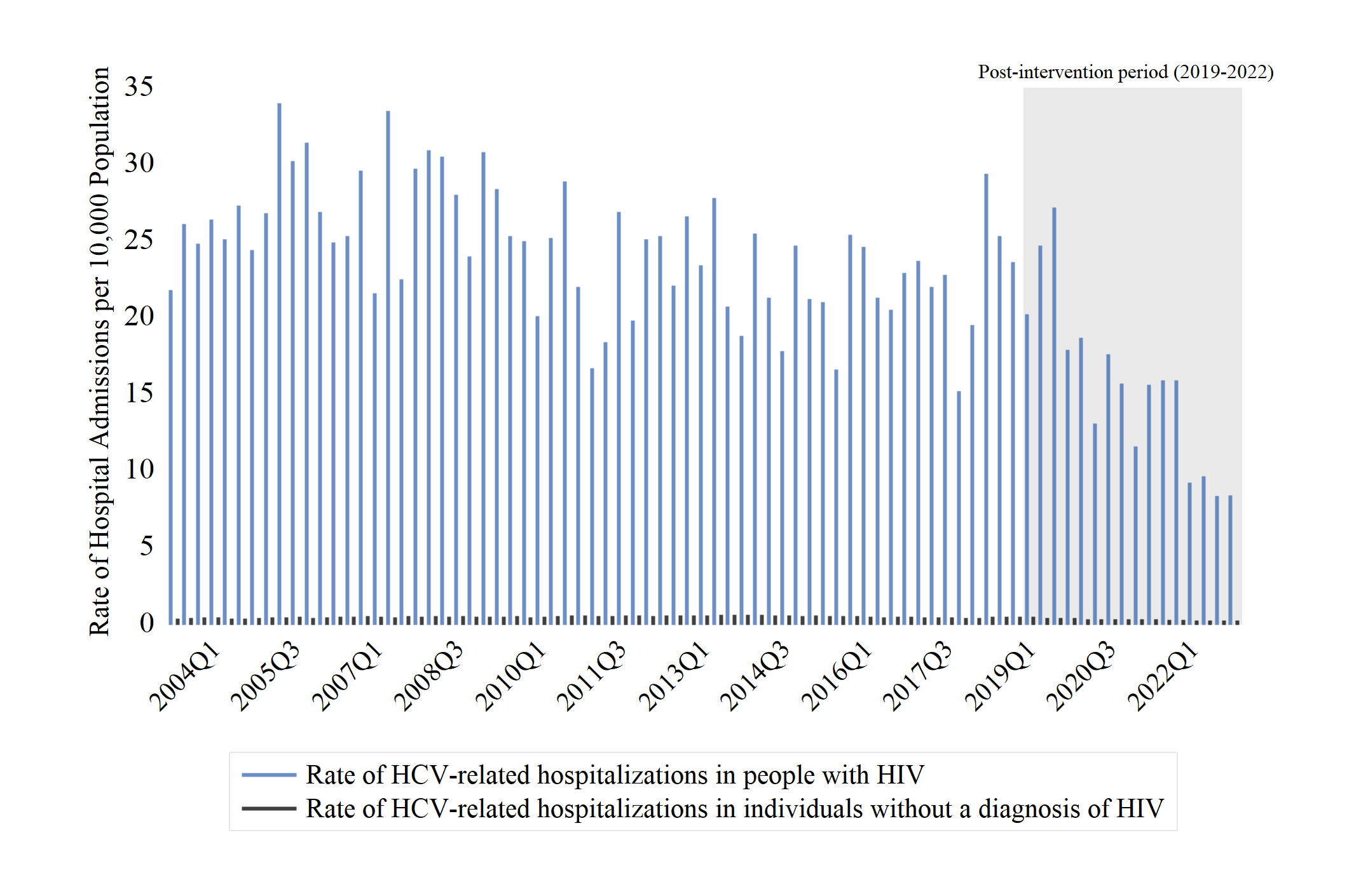
**

**Supplemental Figure 3: Autocorrelation and partial autocorrelation plots for residuals and square of residuals from ARIMA (3,1,0)(0,0,2)[4] model**


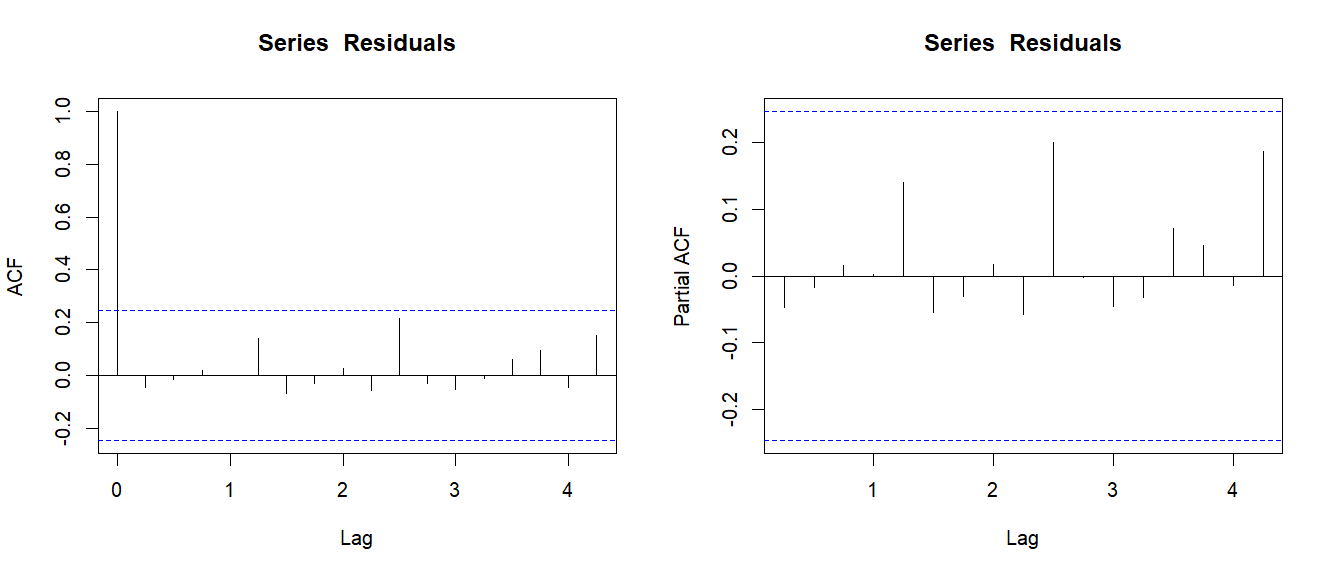


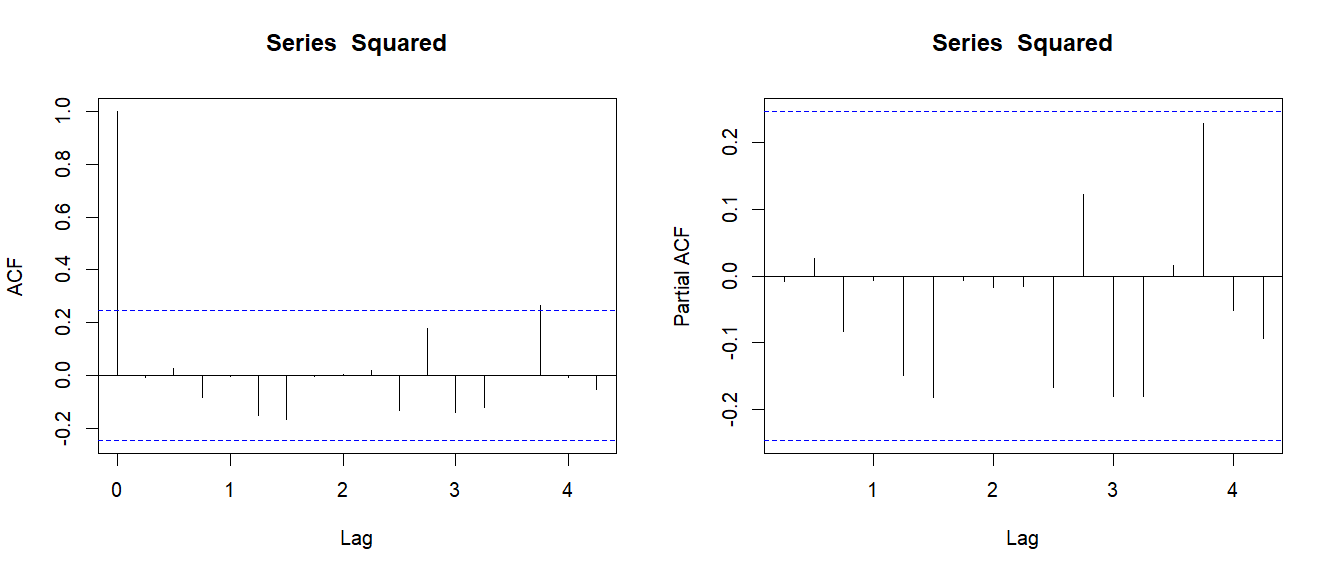

Supplement: ofaf003_Supplementary_Data [file ofaf003_supplementary_data.docx]
